# SARS-CoV-2 infection of salivary glands compromises the production of a secreted antifungal peptide with potential implications for development of oral candidiasis

**DOI:** 10.1371/journal.ppat.1012375

**Published:** 2024-12-12

**Authors:** Areej A. Alfaifi, Tristan W. Wang, Paola Perez, Ahmed S. Sultan, Timothy F. Meiller, Peter Rock, David E. Kleiner, Daniel S. Chertow, Stephen M. Hewitt, Billel Gasmi, Sydney Stein, Sabrina Ramelli, Daniel Martin, Blake M. Warner, Mary Ann Jabra-Rizk

**Affiliations:** 1 Department of Oncology and Diagnostic Sciences, School of Dentistry, University of Maryland, Baltimore, Maryland, United States of America; 2 Department of Restorative and Prosthetic Dental Sciences, College of Dentistry, King Saud bin Abdulaziz University for Health Sciences, Riyadh, Saudi Arabia; 3 King Abdullah International Medical Research Center (KAIMRC), Riyadh, Saudi Arabia; 4 Salivary Disorders Unit, National Institute of Dental and Craniofacial Research, National Institutes of Health, Bethesda, Maryland, United States of America; 5 University of Maryland Greenebaum Cancer Center, University of Maryland, Baltimore, Maryland, United States of America; 6 Department of Anesthesia, School of Medicine, University of Maryland, Baltimore, Maryland, United States of America; 7 Laboratory of Pathology, Center for Cancer Research, National Cancer Institute, National Institutes of Health, Bethesda, Maryland, United States of America; 8 Critical Care Medicine Department, Clinical Center, National Institutes of Health, Bethesda, Maryland, United States of America; 9 Laboratory of Virology, National Institute of Allergy and Infectious Diseases, National Institutes of Health, Hamilton, Montana, United States of America; 10 Genomics and Computational Biology Core, National Institute of Dental and Craniofacial Research, National Institutes of Health, Bethesda, Maryland, United States of America; 11 Department of Microbiology and Immunology, School of Medicine, University of Maryland, Baltimore, Maryland, United States of America; University of California Irvine, UNITED STATES OF AMERICA

## Abstract

Saliva contains antimicrobial peptides considered integral components of host innate immunity, and crucial for protection against colonizing microbial species. Most notable is histatin-5 which is exclusively produced in salivary glands with uniquely potent antifungal activity against the opportunistic pathogen *Candida albicans*. Recently, SARS-CoV-2 was shown to replicate in salivary gland acinar cells eliciting local immune cell activation. In this study, we performed studies to investigate the implications of SARS-CoV-2 infection on salivary histatin-5 production and *Candida* colonization. Bulk RNA-sequencing of parotid salivary glands from COVID-19 autopsies demonstrated statistically significant decreased expression of histatin and amylase genes. *In situ* hybridization, coupled with immunofluorescence for co-localization of SARS-CoV-2 spike and histatin in salivary gland cells, showed that histatin was absent or minimally present in acinar cells with replicating viruses. To investigate the clinical implications of these findings, salivary histatin-5 levels and oral *Candida* burden in saliva samples from three independent cohorts of mild and severe COVID-19 patients and matched healthy controls were evaluated. Results revealed significantly reduced histatin-5 in SARS-CoV-2 infected subjects, concomitant with enhanced prevalence of *C. albicans*. Analysis of prospectively recovered samples indicated that the decrease in histatin-5 is likely reversible in mild-moderate disease as concentrations tended to increase during the post-acute phase. Importantly, salivary cytokine profiling demonstrated correlations between activation of the Th17 inflammatory pathway, changes in histatin-5 concentrations, and subsequent clearance of *C. albicans* in a heavily colonized subject. The importance of salivary histatin-5 in controlling the proliferation of *C. albicans* was demonstrated using an *ex vivo* assay where *C. albicans* was able to proliferate in COVID-19 saliva with low histatin-5, but not with high histatin-5. Taken together, the findings from this study potentially implicate SARS-CoV-2 infection of salivary glands with compromised oral innate immunity, and potential predisposition to oral candidiasis.

## Author summary

Saliva contains antimicrobial peptides part of host innate immunity crucial for protection against colonizing microbial species. Most notable is the antifungal peptide histatin-5 produced in salivary glands cells. SARS-CoV-2 was shown to replicate in salivary gland cells causing tissue inflammation. In this study, we showed decreased expression of histatin genes in salivary glands from COVID-19 autopsies, and co-localization studies of SARS-CoV-2 spike and histatin revealed absence or minimal presence of histatin in acinar cells with replicating virus. To investigate the clinical implications of these findings, we conducted studies using saliva samples from subjects with mild to severe COVID-19, matched with healthy controls. Results revealed significantly reduced histatin-5 in SARS-CoV-2 infected subjects with enhanced prevalence of *C. albicans*. Prospective analysis indicated the decrease in histatin-5 is reversible in mild-moderate disease, and salivary cytokine profiling demonstrated activation of the Th17 inflammatory pathway. The importance of salivary histatin-5 in controlling the proliferation of *C. albicans* was demonstrated using an *ex vivo* assay where *C. albicans* was able to proliferate in saliva with low histatin-5, but not with high histatin-5. Collectively, the findings provide direct evidence implicating SARS-CoV-2 infection of salivary glands with compromised oral innate immunity and predisposition to oral candidiasis.

## Introduction

The oral cavity remains an underappreciated site for SARS-CoV-2 infection despite the evident myriad of oral conditions observed in COVID-19 patients [1–3]. The homeostasis of the oral cavity is maintained by saliva, an extracellular fluid enriched with antimicrobial peptides considered part of the host Th17-type adaptive immune [4]. Most notable is histatin-5 which is unique as it is exclusively produced in acinar cells of salivary glands and exhibits potent antifungal activity against the fungal pathogen *Candida albicans* (*C. albicans*) [5–8]. Although *C. albicans* is a commensal colonizer of the oral cavity, changes in host microenvironment favoring its proliferation allows this opportunistic species to transition into a pathogen causing oral candidiasis (thrush) [9,10]. Therefore, local innate immune defenses and specifically histatin-5 play a central role in maintaining *Candida* in its commensal state [11].

The salivary glands were recently shown to be a target for SARS-CoV-2 infection due expression of viral entry factors in salivary glands epithelial cells [12,13]. A recent study by Huang and Perez *et al*. (2021) [14] demonstrated that salivary gland acinar cells are robust sites for SARS-CoV-2 infection and replication, eliciting local immune cell activation. These findings indicate that the source of virus in saliva is likely derived from infected cells in the salivary glands [14]. In fact, cases of inflamed salivary glands have been reported in COVID-19 patients [15,16]. Importantly, SARS-CoV-2 infected subjects have been reported to develop superimposed infections, including oral candidiasis [2,17]. However, while SARS-CoV-2 infects salivary glands, it is not known if the risk of oral candidiasis is due to viral effects on acinar cell expression of histatins. To that end, we performed mechanistic and clinical studies to investigate the implications of SARS-CoV-2 infection of salivary glands on oral innate immune defenses and *Candida* colonization. The findings establish the oral cavity as a robust site for SARS-CoV-2 infection warranting reassessment of the risks for oral opportunistic infections in COVID-19 patients.

## Results

### SARS-CoV-2 infection of parotid glands affects expression of histatins

**Fig 1:**
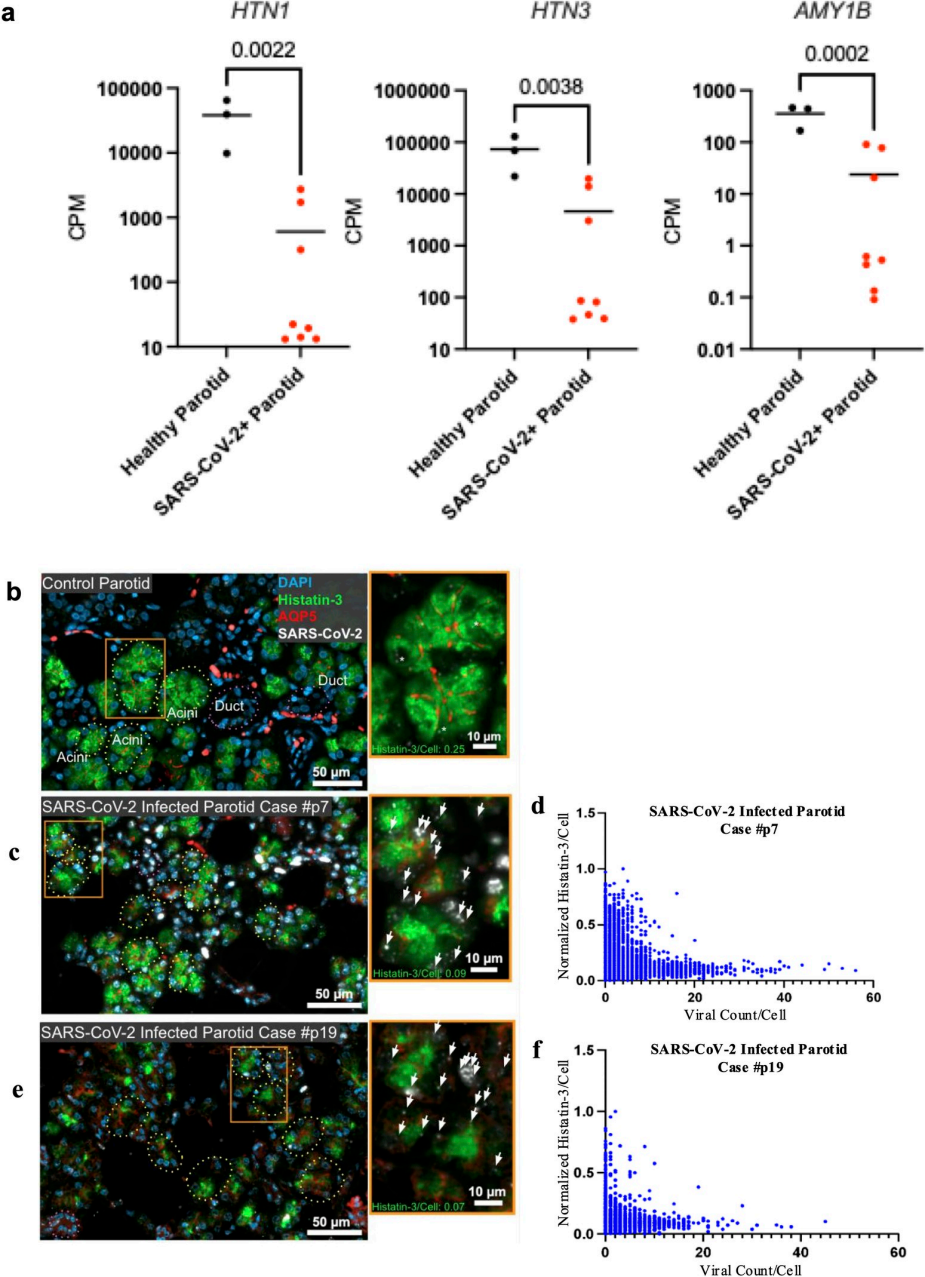
Impact of SARS-CoV-2 salivary gland infection on histatin gene expression and production. **(a)** Parotid expression of histatin (*HTN1* and *HTN3*) and amylase (*AMY1B*) genes from deceased COVID-19 subjects (n = 8) and healthy control subjects (n = 3) using bulk RNA sequencing. **(b-f)** Co-localization studies using *in situ* hybridization for SARS-CoV-2 (*white dots* and *arrows*) and immunofluorescence for histatin-3 (*green*) with quantitative correlation between the intensity of histatin and SARS-CoV-2 counts within infected acinar cells of parotid tissue from **(b)** healthy subject (*white asterisk*, non-specific signal) and **(c, e)** deceased COVID-19 patients. Acini (dotted circles) identified based on expression of AQP5 (*red*, apical membrane); *inset* (*orange box*) shows the presence of the viral genome in acinar cells of the parotid glands. **(d, f)** Per acinar cell expression of histatin and the per cell viral count are inversely proportional as shown in two representative COVID-19 cases (P7, P19).

Bulk RNA-sequencing data from infected parotid glands tissue from deceased COVID-19 patients and uninfected control tissue demonstrated significantly reduced expression of histatin genes (*HTN1*, *HTN3*) (>100-fold) (Fig 1A). Confirmatory hybrid *in situ* hybridization and immunofluorescence microscopy co-localization studies of SARS-CoV-2 and histatins, respectively, demonstrated significant (p<0.001) reduction of histatin protein expression in virus infected parotid gland acinar cells with histatin intensity inversely proportional to viral count (Fig 1B–1F) (S1 Fig). Importantly, gene expression of the salivary marker amylase (*AMY1B*) exhibited a similar trend to that of histatin genes.

### SARS-infected patients secrete significantly less histatins potentiating oral candidiasis

**Fig 2:**
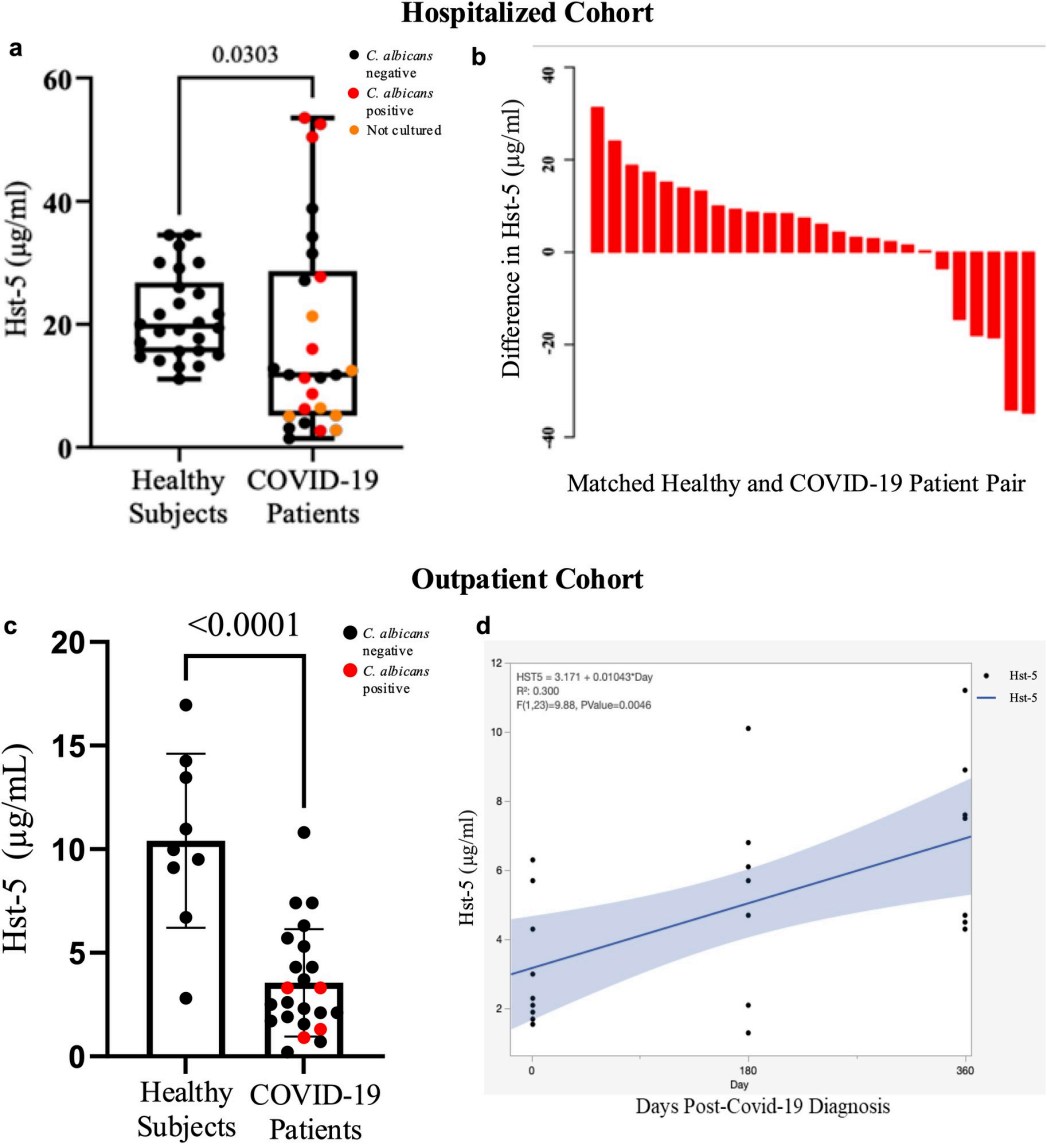
Salivary histatin-5 (Hst-5) levels and fungal colonization in (a, b) prospectively sampled hospitalized and (c, d) outpatient COVID-19 cohorts. **(a)** Boxplot with Hst-5 concentrations and *Candida* recovery from 26 saliva samples from hospitalized COVID-19 patients and matched healthy controls. **(b)** Waterfall plot depicting 26 pairs of hospitalized COVID-19 patients and healthy subjects matched for race and age. Bar height represents differences among pairs in Hst-5 levels (μg/ml) between healthy controls and COVID-19 patients. **(c)** Bar plot depicts Hst-5 concentrations and *Candida* recovery of 9 healthy subjects and 23 COVID-19 outpatients’ saliva. Red marked dots indicate cultured saliva contained candidal outgrowth **(d)** Linear regression analysis of serially sampled COVID-19 outpatients’ saliva (n = 8) shows time-dependent restoration of Hst-5 concentration from the post-acute phase to the chronic phase, based on longitudinally collected samples from acute phase (3–15 days) to chronic phase (6–12 months). Blue line, linear fit; light blue shading, confidence interval of the linear fit.

Clinical studies were conducted using two COVID-19 patient cohorts (hospitalized and outpatient), with ranging disease severity and matched healthy controls. Significant differences in histatin-5 concentrations in clarified saliva were seen where the average for the control group was 21.3μg/ml and 18.4μg/ml for the COVID-19 group. The difference in concentrations was also seen in age-and-race-matched comparison between each COVID-19 patient and their matched control (Fig 2B). Strikingly, 14 of the COVID-19 patients had concentrations below 10μg/ml (Fig 2A). *Candida* colonization was assessed by culture; where no *Candida* was recovered from any of the controls, 47% of tested COVID-19 patients were positive for *C. albicans*, some heavily colonized (Fig 2A). Histatin-5 concentrations were also significantly (3.6 vs 10.4 μg/ml) lower in whole saliva from a prospectively sampled outpatient cohort (Fig 2C). Prospective analysis of serial samples indicated a statistically significant trend in increase in histatin-5 concentration from the post-acute to the chronic phase (Fig 2D). No statistical differences (p-values > 2.0) in histatin-5 concentrations were seen between subjects based on disease severity or other variables. Histatin-5 concentrations are expected to differ in clarified vs. unclarified saliva.

### Significant changes in histatin-5 concentrations over the course of COVID-19 disease in prospectively sampled subjects

**Fig 3:**
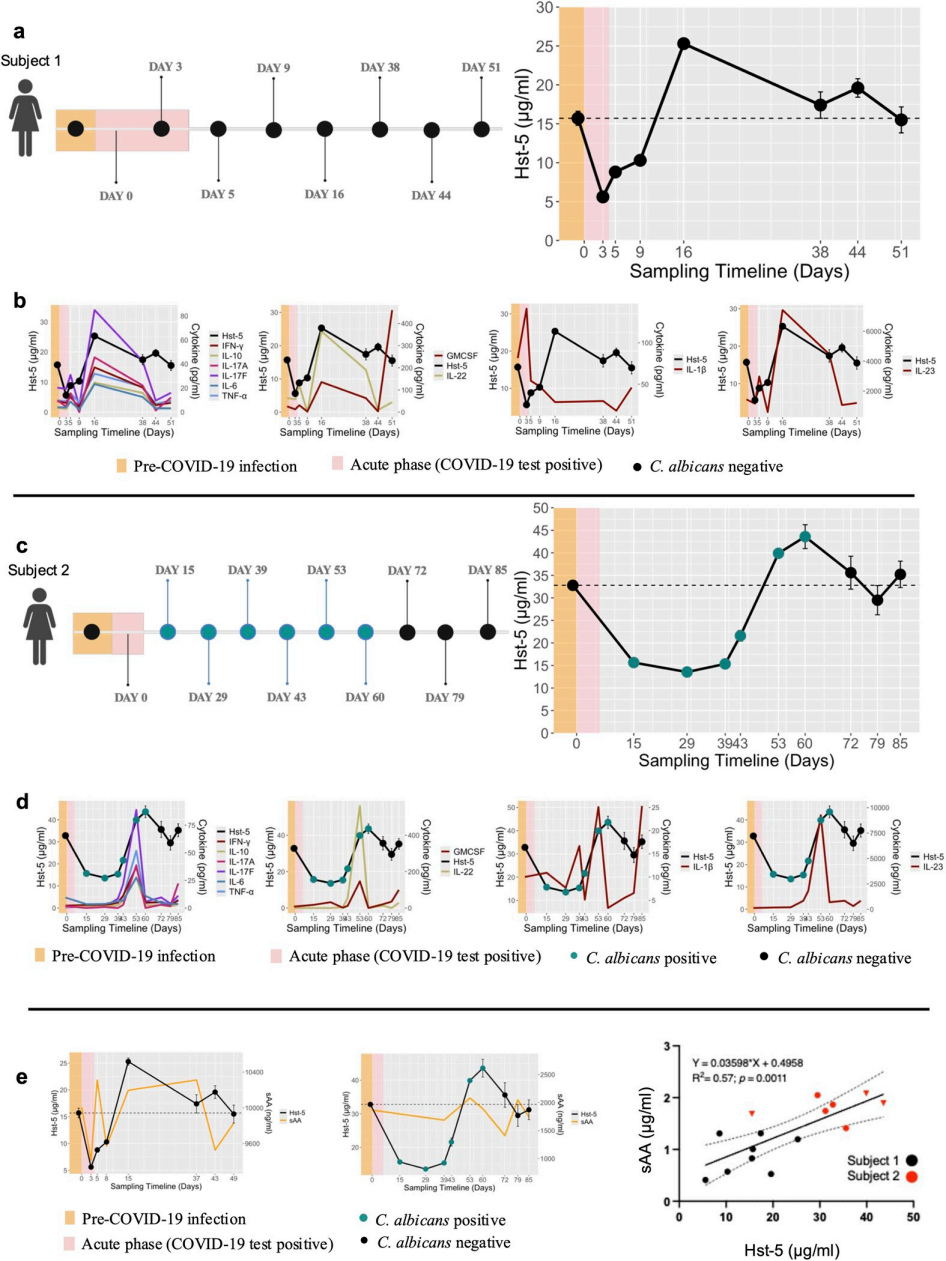
Prospective time course longitudinal analysis of saliva samples for Hst-5 and salivary α-amylase (sAA) from two subjects with moderate COVID-19. Changes in Hst-5, sAA and cytokine concentrations in two longitudinally sampled subjects prior to and during the acute and post-acute phases of COVID-19 disease. Timeline of sampling during COVID-19 infection up to 49 and 85 days post-COVID-19 infection for subjects 1 and 2 **(a** and **c,** respectively**)**. Measurement of Hst-5 concentrations and fungal culturing of samples from the two subjects prior to and during the acute and post-acute phases of COVID-19 disease. Line graphs in bottom rows depict similar trends for Hst-5 and Th17 associated cytokine levels for subjects 1 and 2 **(b** and **d,** respectively**)**. Upon culturing, *C. albicans* was recovered from the initial 6 samples from subject 2 **(c, d)**. Prospective time course analysis of salivary amylase (sAA) concentrations with respect to histatin-5 prior to and during the acute and post-acute phases of COVID-19 disease. Green dots denote positive *C. albicans* samples **(e)**. Scatterplot depiction of correlation test of histatin-5 and amylase (sAA) levels in saliva samples from subjects 1 and 2. Triangles denote positive *C. albicans* samples **(f)**. Created using Biorender.com.

A total of 5 subjects with mild-moderate disease were prospectively sampled to monitor changes in histatin-5 concentrations. For two of the subjects sampled up to 49 and 85 days, respectively, histatin-5 concentrations were measured prior to COVID-19 infection. For subject 1, histatin-5 prior to COVID-19 disease was 15.7μg/ml; however, in sample recovered during the acute phase, concentration was 5.6μg/ml (64.3% drop), gradually increased during the post-acute phase with a spike noted on Day 15 (25.3μg/ml) returning to baseline level in the last sample analyzed (Fig 3A). For subject 2, histatin-5 concentration prior to COVID-19 was 32.8μg/ml and 15.7 μg/ml (52.3% drop) in the sample recovered during the post-acute phase; concentration gradually increased over subsequent days with a spike noted on Day 60 (43.6μg/ml) before returning to pre-COVID-19 levels on last day sampled (Fig 3C). Significantly, *C. albicans* was recovered from the initial 6 samples of subject 2, but not from the last 3 samples that followed restoration of histatin-5 to pre-COVID-19 level (Fig 3C). Additionally, amylase was selected as a salivary protein marker and measured concentrations indicated a similar trend to that for histatin-5 in saliva samples of both subjects (Fig 3E and 3F). Three additional subjects were also prospectively sampled; histatin-5 concentrations in first samples during acute phase were 2.4, 4.1 and 5.6μg/ml, respectively which increased during the post-acute phase to 10.9, 13.2, and 12.5μg/ml, respectively in the last samples tested (S2 Fig). No *Candida* was recovered from any of these samples.

### Activation of Th17 inflammatory pathway concomitant with changes in histatin-5 concentrations

Cytokine profiling on saliva from subjects 1 and 2 demonstrated a similar trend to histatin-5, where compared to pre-COVID-19 samples, a notable increase in the Th17-associated inflammatory cytokines was seen gradually decreasing to baseline levels (Fig 3B and 3D). Interestingly, in both subjects, the spike in cytokine concentrations coincided with salivary histatin-5 recovery, and the clearance of *C. albicans* in subject 2 (Fig 3C). Changes in Th17-associated inflammatory cytokines were similarly observed during the post-acute phase for subject 3 (S2 Fig).

### *C. albicans* proliferates in saliva from COVID-19 positive subjects with low histatin-5

**Fig 4:**
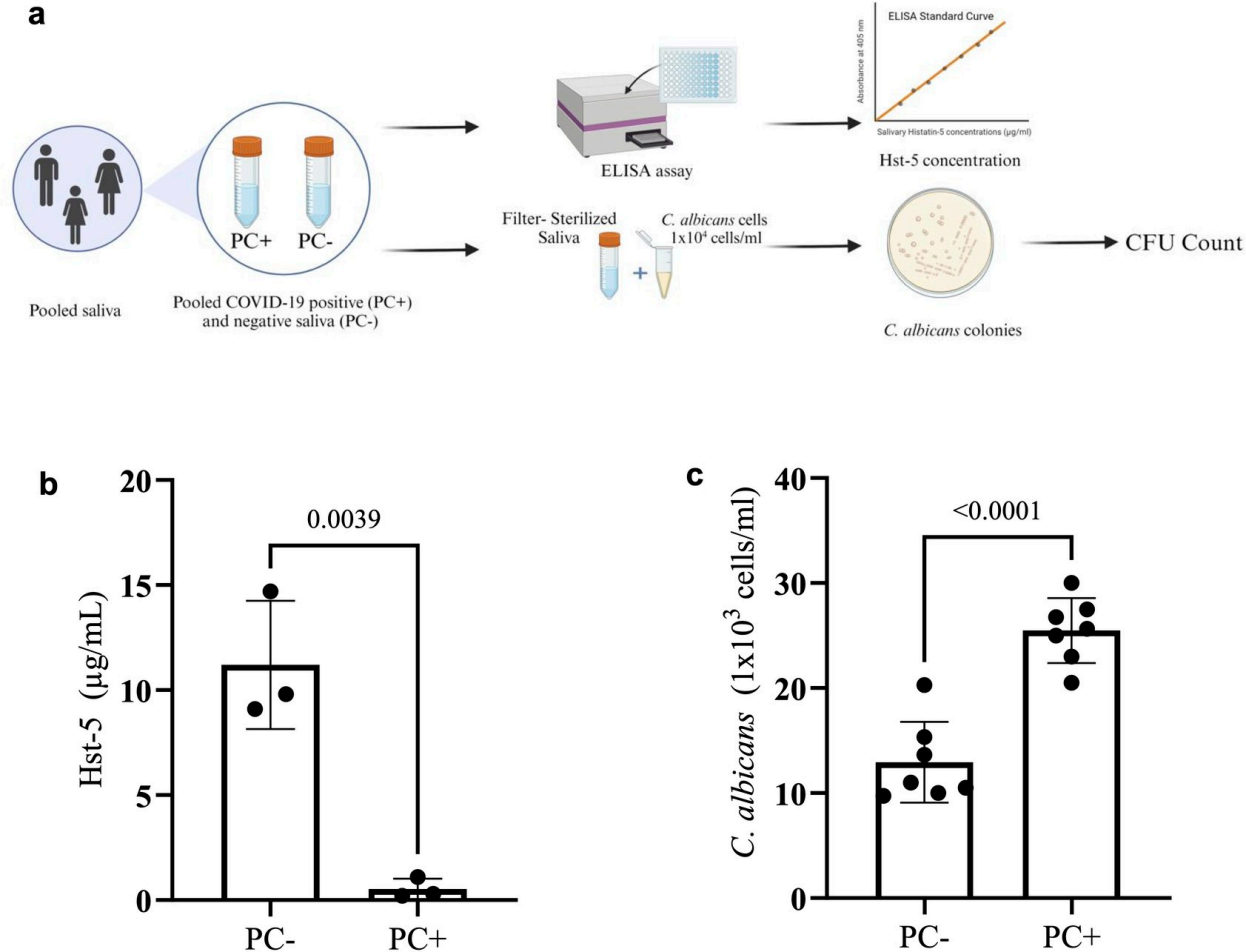
*Ex vivo* proliferation assay using pooled saliva. **(a)** Workflow for histatin-5 measurement and proliferation assay using pooled saliva samples from 3 subjects under a COVID-19 infected state (PC+) and recovered state (PC-). **(b)** Bar plots depict Hst-5 concentrations (μg/ml) from pooled saliva samples; **(c)** recovered *C. albicans* (cells/ml) following 1 h incubation in pooled saliva samples seeded with 1x10^4^ cells/ml of *C. albicans*. Created using Biorender.com.

*Ex vivo* anti-candidal assay was used to assess proliferation of *C. albicans* in pooled saliva from subjects during acute COVID-19 (0.5μg/ml histatin-5) and after recovery (11.5 μg/ml histatin-5) (Fig 4A and 4B). Based on *C. albicans* CFUs, an average of 2.6x10^4^ cells/ml *C. albicans* was recovered from the low histatin-5 sample, and 1.29x10^4^ cells/ml from the high histatin-5 sample indicating that COVID-19 low histatin-5 saliva allowed *C. albicans* to proliferate significantly (Fig 4C).

## Discussion

Innate immunity not only represents the first line of defense providing the initial host response to pathogens, but also activates the adaptive immunity to establish and maintain tissue homeostasis [11]. The host-produced salivary histatin-5 is considered to play a vital role in innate immune defenses against microbial species, primarily *C. albicans*, the most common opportunistic fungal pathogen [4,6]. The recent demonstration of SARS-CoV-2 replication in salivary glands acini cells where histatins are produced indicate that histatin production may be compromised in infected individuals. Comparative transcriptional analysis of SARS-CoV-2 infected salivary gland tissue demonstrated significant downregulation in expression of histatin genes. Importantly, co-localization studies demonstrated an inversely proportional association between histatin and viral counts in acinar. The clinical implications of these findings were revealed using various cohorts of SARS-CoV-2 infected subjects where overall, significant reduction in histatin-5 levels was seen in infected subjects compared to healthy controls. Prospective sampling of moderately symptomatic COVID-19 subjects allowed us to glean some insights into the temporal relationship between infection and histatin expression. Although findings indicated that the decrease in histatin-5 during COVID-19 is likely reversible, based on inversely proportional association between histatin and viral counts in severe COVID-19, damage to salivary gland function may be irreparable, and affected individuals may remain predisposed to oral candidiasis as part of the Long COVID syndrome [18]. We acknowledge limitations to the clinical studies in excluding confounding host factors that may influence histatin-5 or predispose to oral candidiasis such as steroids or medications. However, it is notable that histatin-5 levels were decreased in all patient cohorts, with no associations seen between histatin-5 concentrations, subject demographics, disease severity or *Candida* carriage. Significantly, evaluation of amylase gene expression and concentration clearly indicates that SARS-CoV-2 infection likely impairs salivary gland function and suppresses host salivary transcriptome and secreted proteome.

It is interesting that histatin-5 concentrations were concomitant with activation of the Th17 inflammatory pathway, which is not surprising as antimicrobial peptides, including histatins, are part of the host Th17-type adaptive immune response [11,19,20]. In fact, IL-17 was shown to induce histatins in cultured salivary gland cells [21]. Since in the tissues of the oral cavity, the IL-17/Th17 signaling pathway is essential for host protection against *C. albicans* infection [19,20,22], it was also not surprising that activation of Th17 pathway coincided with clearance of *C. albicans* from the colonized subject. Finally, the importance of histatin-5 in controlling the proliferation of *C. albicans* was demonstrated when saliva with low histatin-5 allowed *C. albicans* proliferation *in vitro*. These findings provide experimental evidence establishing the importance of histatin-5 in maintaining *Candida* in the commensal state. Collectively, our findings provide mechanistic insights underscoring the oral cavities’ diverse susceptibilities to SARS-CoV-2 infection, prompting a reassessment of oral opportunistic infection risks and their potential long-term impacts on oral health.

## Methods

### Ethics statement

*NIH*. Autopsies are exempt from NIH single institutional review board (IRB), consent from families of fatal COVID-19 cases was obtained. Otherwise, NIH single IRB conducts ethical reviews for human research studies as required by Department of Health and Human Services regulations for the Protection of Human Subjects. All patients seen at the author’s (B.M.W.) institute the National Institutes of Health/National Institute of Dental and Craniofacial Research (NIH/NIDCR) reported herein provided informed written consent before participation in IRB-approved research protocols (NIH IRB: 20-D-0094, NCT04348240; NIH IRB: 15-D-0051, NCT02327884). Individuals on 20-D-0094 had the option to receive a $50 payment per visit ($300 total) to offset the cost of travel.

### Bulk RNA-sequencing analysis of salivary gland tissue

Total RNA was extracted from parotid glands at autopsy from COVID-19 subjects and from uninfected donors. Bulk RNA-sequencing was performed using Illumina platform and gene expression levels of histatin (*HTN1* and *HTN3)* and amylase (*AMY1B)* were compared.

### Co-localization of SARS-CoV-2 and histatin in salivary gland tissue using *in situ* hybridization and immunofluorescence, respectively

Studies were performed on parotid biopsies from uninfected donors and from COVID-19 subjects at autopsy with high and low copy virus number [14,23]. Tissue samples were probed with anti-histatin antibody and hybridized with V-nCoV2019-S antisense probe targeting spike protein sequence of viral RNA. Visiopharm software was used for imaging and an algorithm was designed to detect and count virus and quantify histatin intensity per cell.

### Study subjects and clinical samples

Study population included: *Hospitalized cohort*. Adult hospitalized COVID-19 positive patients (n = 26) and a matched control (age, race and gender) group of healthy healthy volunteers (n = 26). *Outpatient cohort*. 33 subjects with mild-moderate disease and a total of 68 prospectively collected samples. *Prospective cases*. 5 otherwise healthy outpatient subjects prospectively sampled during the course of COVID-19 disease (29 samples). Institutional Review Board (IRB) approval and informed written consent was obtained. Saliva samples (histatin-5 and cytokines) and oral swabs were collected from participating subjects as we previously described using Salivette saliva clarification system (hospitalized cohort and controls) and whole saliva (outpatient cohort and controls) [14,24,25]. Oral samples were cultured on fungal media to evaluate *Candida* growth and chromogenic medium was used for speciation.

### Measurement of histatin-5 salivary levels

A standard ELISA was performed as we previously described [24] using a histatin-5 specific rabbit polyclonal antibody. Samples were tested in triplicate on two separate occasions and average histatin-5 concentration calculated in μg/ml.

### Salivary cytokines and amylase profiling

Cytokines were analyzed using the Luminex Multianalyte System in triplicate and results expressed in pg/ml. Salivary α-amylase (sAA) concentration was measured in duplicate using the Human Salivary Amylase ELISA kit (Novus Biologicals) as per manufacturer instructions and average of results expressed in ng/ml.

### *Ex vivo* salivary *C. albicans* inhibition assay

Pooled saliva samples were recovered from subjects pre- and post-COVID-19 to generate a COVID-19 positive and a COVID-19 negative sample. Samples were inoculated with *C. albicans* cells (1×10^4^ cells/ml) and following 1 h incubation at 37°C aliquots were plated on YPD agar for colony forming units (CFU) counts (cells/ml) (Fig 4A).

### Statistical analysis

*Wilcoxon rank-sum*: to compare histatin-5 levels; *t-test*: to evaluate associations between histatin-5 and patient characteristics; *unpaired two-sample t-tests*: for *in vitro* assays; *ANOVA analysis with Bonferroni correction for multiple measurement*: *HTN1*, *HTN3* and *AMY1B* expression levels. Correlation between histatin and amylase was determined using Pearson’s correlation test. Figures were constructed using GraphPad Prism 10.2.1 and R statistical programming software.

## Supporting information

S1 Fig *In situ* hybridization and immunofluorescence controls. **(a)** Immunofluorescence (IF) and *in situ* hybridization (ISH) controls (see also **Fig 1**). Positive and negative controls for IF, antibody anti-Hst-3 in control parotid shows strong IF (green) in acini structures and no signal is observed in ducts. The isotype control shows no signal. **(b)** Probe anti-human *PPIB* shows strong signal in all the cells (white); negative control probe shows sparse, rare positive signal in the tissue.
(TIFF)

S2 Fig Prospective sampling and analysis of saliva from three subjects with moderate COVID-19. Timeline of sampling and measurement of salivary Hst-5 and cytokine concentrations in samples recovered during the acute phase of the disease and upon recovery. Table presents cytokine values measured in the first and last samples recovered from the three subjects and the percent change in levels between the samples. Created using Biorender.com
(TIFF)

S1 Data Raw data for Figs 1, 2, 3, 4 and S2. (XLSX)
